# Evolution of Lower Brachyceran Flies (Diptera) and Their Adaptive Radiation with Angiosperms

**DOI:** 10.3389/fpls.2017.00631

**Published:** 2017-04-24

**Authors:** Qingqing Zhang, Bo Wang

**Affiliations:** ^1^State Key Laboratory of Palaeobiology and Stratigraphy, Nanjing Institute of Geology and Palaeontology, Chinese Academy of SciencesNanjing, China; ^2^University of Science and Technology of ChinaHefei, China; ^3^Key Laboratory of Zoological Systematics and Evolution, Institute of Zoology, Chinese Academy of ScienceBeijing, China

**Keywords:** brachyceran flies, angiosperm, mid-Cretaceous, pollinator, co-evolution

## Abstract

The Diptera (true flies) is one of the most species-abundant orders of Insecta, and it is also among the most important flower-visiting insects. Dipteran fossils are abundant in the Mesozoic, especially in the Late Jurassic and Early Cretaceous. Here, we review the fossil record and early evolution of some Mesozoic lower brachyceran flies together with new records in Burmese amber, including Tabanidae, Nemestrinidae, Bombyliidae, Eremochaetidae, and Zhangsolvidae. The fossil records reveal that some flower-visiting groups had diversified during the mid-Cretaceous, consistent with the rise of angiosperms to widespread floristic dominance. These brachyceran groups played an important role in the origin of co-evolutionary relationships with basal angiosperms. Moreover, the rise of angiosperms not only improved the diversity of flower-visiting flies, but also advanced the turnover and evolution of other specialized flies.

## Introduction

The Diptera (true flies) is one of the most species-abundant orders of Insecta, and they are certainly one of the most ecologically ubiquitous and significant orders of insects (Grimaldi and Cumming, 1999). They are among the most ancient pollinators of flowering plants (Bernhardt and Thien, 1987; Labandeira, 1998), and played an important role in the origin of co-evolutionary relationships with flowering plants and insects (Thien et al., 2000; Ssymank et al., 2008).

The Cretaceous is a time of important developments in angiosperms that angiosperms rose to dominance during the Albian-Cenomanian, and become forest dominants during the Campanian-Maastrichtian (Friis et al., 2010; Peralta-Medina and Falcon-Lang, 2012). Although the rise of Angiosperms did not generate an immediate increase in insect diversification within major insect groups based on Bayesian fossil-based analyses, but the influence of the radiation of Angiosperms on insect diversification is not excludable (Condamine et al., 2016). The angiosperm radiations provided new food resources and habitats, and had a profound effect on flies, beetles, and other insects (Wang et al., 2013). The interval since the middle Early Cretaceous to early Late Cretaceous witnessed the significant transformation to the modern terrestrial world, between this time (from 125 million years ago to 90 million years ago), and there were significant shifts in the major ecological associations among plants, insects, and other organismic groups dominant on land (Labandeira, 2010).

Brachyceran flies are quite abundant during Mesozoic, especially from the Middle-Late Jurassic to mid-Cretaceous. The middle Early Cretaceous to the early Late Cretaceous is a significant period for brachyceran flies, including the ecological success of some flower-visiting flies and extinction of several important groups, such as Eremochaetidae and Zhangsolvidae (Arillo et al., 2015; Zhang et al., 2016a). The extant family Tabanidae, Nemestrinidae, Bombyliidae are among the commonest pollinators of most extant basal angiosperms, and their early evolution are important for understanding the co-evolution between flies and angiosperms. The probable impact of floristic changes on brachyceran flies during the Early Cretaceous has been widely accepted, but supporting fossils are still relatively few (Grimaldi, 1999; Labandeira and Currano, 2013). Recently abundant Cretaceous fossils have been described and our knowledge about the evolution of brachyceran flies has improved greatly (e.g., Grimaldi, 2016; Zhang et al., 2016a,b). In this paper, we review the fossil record and early evolution of five groups, and briefly discuss their probable ecological associations with early angiosperms.

## Fossil Record

### Tabanidae

**FIGURE 1:**
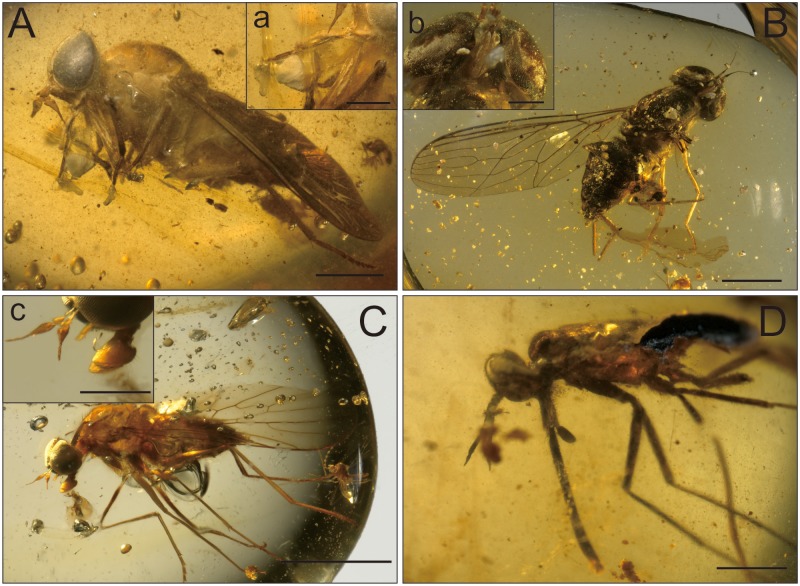
**Four types of mouthparts in mid-Cretaceous Burmese amber. (A)** Tabanidae, scale bar = 2 mm; **(a)** Mouthparts, scale bar = 1 mm. **(B)** Nemestrinidae, scale bar = 2 mm; **(b)** Mouthparts, scale bar = 0.5 mm. **(C)** Bombyliidae, scale bar = 2 mm; **(c)** mouthparts, scale bar = 0.5 mm. **(D)** Zhangsolvidae with a long proboscid, scale bar = 1 mm.

Tabanidae, normally called horse flies or deer flies, is an ubiquity family, and the most diverse family-level clade that has more than 4000 species distributed in 156 genera worldwide (**Figure 1A**; Pape et al., 2011). They are stout-bodied flies, with larger first flagellomere and 4-8 apical flagellomeres; legs with two apical spurs on midtibia, tarsi with pulvilliform empodium; wing venation with R4 and R5 enclose wing apex, form a large ‘Y’ across the wing tip; cell br, bm and d large, cell cup closed near wing margin; calypters almost always well developed (Colless and McAlpine, 1991; Burger, 2009). Tabanidae is type family of Tabanidae which characteristiced by the presence of a venom canal of the larval mandible (Kerr, 2010; Morita et al., 2016).

Tabanids are relatively scarce in the fossil record, but in Cenozoic, they are quite abundant as fossil recorded from Miocene of Florissant, from North American, Germany, French, and Switzerland Oligocene, from England and Baltic amber Eocene/Oligocene, Pliocene from Europe and Africa (Martins-Neto, 2003). The oldest record of a true tabanid was reported from the Lower Cretaceous Durlston Formation of England. Till now, five species of tabanids was recorded in the Early Cretaceous and one species primitively in Therevidae was moved to the tabanid genus *Cratotabanus* Martins-Neto and Santos (1994; Ren, 1998; Martins-Neto, 2003; Mostovski et al., 2003; Zhang, 2012). Fossils from the Late Cretaceous are quite rare, with only one species and genus from Late Cretaceous of New Jersey amber, together with two newly described species in Burmese amber (Grimaldi et al., 2011; Grimaldi, 2016). Flower-feeding tabanids (Pangoniinae) appear at least in the Early Cretaceous (Martins-Neto and Santos, 1994; Ren, 1998; Zhang, 2012). A recent molecular analysis calibrated using several key fossils support that the divergence of Tabanidae and their sister clade Athericidae, in the Early Cretaceous, approximately 135 Ma (Morita et al., 2016).

### Nemestrinidae

Nemestrinidae commonly called tangle-veined flies, is cosmopolitan but quite a small group of brachycerous flies, with about 300 extant species in over 20 genera (**Figure 1B**; Bernardi, 1973; Mostovski and Martínez-Delclòs, 2000). They are usually medium-sized flies with body stout and dense pilosity, wings are usually longer than body (Wedmann, 2007; Woodley, 2009). They can be easily recognized by a so-called diagonal vein, the compound diagonal vein obliquely aligned through the wing; they also have some characteristics including tibiae without apical spurs, empodium pulvilliform, and one segmented cercus and flagellum often formed into a slender stylus (Yeates, 1994; Wedmann, 2007). Fossil tangle-veined flies are quite abundant since Mesozoic, many nemestrinids were found in Late Jurassic and Early Cretaceous, and some Cenozoic nemestrinids were described, mainly from the Oligocene of Florissant, USA. Ansorge and Mostovski (2000) listed an updated list of all taxa of Nemestrinidae, and additional taxa have been described from the Eocene of Germany (Wedmann, 2007), mid-Cretaceous Burmese amber (Grimaldi, 2016; Zhang et al., 2017), and a doubtable genus without diagonal vein from the Late Jurassic of China (Zhang et al., 2008). The oldest fossil nemestrinids are from the Late Jurassic of Karabastau, Kazakhstan (Rohdendorf, 1968; Mostovski, 1998). Ansorge and Mostovski (2000) hypothesized that the family Nemestrinidae probably originated in the Late Triassic or Early Jurassic, as the oldest fossil Nemestrinidae appeared in Early Jurassic and fossil nemestrinids demonstrate a high taxonomic diversity since the Middle-Late Jurassic. Nemestrinidae is thought to be a sister group of Apioceridae in Nemestrinoidea supported by their parasitic larval lifestyle (Woodley, 1989; Yeates, 2002).

### Bombyliidae

Bombyliidae (bee flies) is a quite diverse and widely distributed family of Asiloidea. It is a cosmopolitan group and a quite large family that comprising over 4500 described extant species around the world (**Figure 1C**; Evenhuis, 1994; Evenhuis and Greathead, 2003; Wedmann and Yeates, 2008). They are commonly robust flies, often with long projecting proboscis and usually densely hairs (Colless and McAlpine, 1991; Greathead et al., 2009). They feed on nectar as well as pollen, many of them using a long proboscis to probe flowers (Grimaldi, 2016). The fossil of adult bee flies can be distinguished by the following features: antenna usually with flagellomere coniform, usually with one or two flagellomeres and a terminal bristlelike stylus; wing R2+3 and R4 usually strongly curved distally, meeting costa at about a right angle; R4+5 branched, R4 and R5 usually encompass wing tip; three (rarely two) posterior cells (Greathead et al., 2009). Fossil bee flies are quite abundant in Cenozoic, especially in the Oligocene and Eocene. Till now, about 70 species in about 40 genera have been described from Florissant of USA, France, Germany, and Dominican and Baltic ambers. The fossil record of Bombyliidae has been reviewed by Hull (1973) and Evenhuis (1994), and new taxa was recently described by Nel and De Ploëg (2004), Nel (2006), and Wedmann and Yeates (2008).

Grimaldi (2016) suggested that the radiation age of Bombyliidae is the Late Cretaceous, but Lamas and Nihei (2007) suggested a Middle Jurassic age based on the molecular phylogenetic analysis. Molecular models and biogeography support a Late Mesozoic diversification of asiloids, with Bombyliidae at the base of the Asiloidea (Winterton et al., 2015; Grimaldi, 2016). Unambiguous Mesozoic bombyliids are extremely rare. Recently, some definitive new records of Bombyliidae in mid-Cretaceous Burmese amber show that bombyliids have already diversified, and these fossils provide new insights into the early evolution of Cretaceous bee flies (Shi et al., 2012; Grimaldi, 2016; Zhang et al., 2016b).

### Eremochaetidae

Eremochaetidae is a Mesozoic extinct family that was established by Ussatchov based on two species in two different genera (Ussatchov, 1968). Eremochaetidae is a quite rare family that was found only in Late Mesozoic, mainly in Early Cretaceous. Till now, only 15 species in nine genera have been described in China, Kazakhstan, Mongolia, Russia and Burmese amber (Ussatchov, 1968; Kovalev, 1989; Ren and Guo, 1995; Mostovski, 1996; Ren, 1998; Zhang, 2014; Zhang et al., 2014, 2016b). All eremochaetids have the characters: eyes very large, occupying the greater part of the head; thorax short and convex; Sc is stout, R1 is very long; cross-vein is absent, causing the vein R4+5 (sometimes R2+3 and R4+5) to arise from cell d; the ovipositor is needle-shaped in all female eremochaetids (Ussatchov, 1968; Zhang et al., 2014). Zhang (2014) described and illustrated the structures of the male genitalia for the first time, and reasoned that these characteristics probably represent the base type of the primitive lower Orthorrhapha of Brachycera. The latest occurrence of eremochaetids is from the mid-Cretaceous Burmese amber (Zhang et al., 2016a). The highly developed, hypodermic-like ovipositor and enlarged tridactylous characteristic in pretarsus supported their endoparasitoid life, and their primitive mouthparts were probably used to feed on nectar (Grimaldi and Barden, 2016; Zhang et al., 2016a). Eremochaetidae is probably related to the superfamily Archisargoidae based on the morphological characteristics (Grimaldi and Barden, 2016). The fossil record of eremochaetids reveals that the extinction of these ancient parasitoids probably occurred by the end of the Late Cretaceous and coincided with the rise of angiosperms, perhaps owing to competition from newly evolved parasitoid wasps and flies which extant ones are mostly flower-visiting insects (Eggleton and Belshaw, 1993; Feener and Brown, 1997; Gilbert and Jervis, 1998; Zhang et al., 2016a).

### Zhangsolvidae

The Zhangsolvidae is an extinct family of brachyceran flies that erected by Nagatomi and Yang (1998) for the genus *Zhangsolva cupressa* found in the Early Cretaceous Laiyang Formation (**Figure 1D**; Zhang et al., 1993; Nagatomi and Yang, 1998; Arillo et al., 2015). Zhangsolvidae is a quite rare family that till now six species in four genera that found only in Cretaceous: five species and three genera in Early Cretaceous of China, Spain, Brazil and one species and genus in Late Cretaceous Burmese amber (Zhang et al., 1993; Nagatomi and Yang, 1998; Mazzarolo and Amorim, 2000; Wilkommen and Grimaldi, 2007; Arillo et al., 2015). Zhangsolvidae has a stout body, with very long and quite slender proboscis, vein M1 strongly arched, M3 fused to M4 and CuA fused to CuP (Nagatomi and Yang, 1998; Arillo et al., 2015). The placement of Zhangsolvidae is within Stratiomyomorpha supported by the presentation of phylogenetic analysis of 52 morphological characters for 35 taxa (Arillo et al., 2015). New zhangsolvids specimens from Early Cretaceous Spanish amber and mid-Cretaceous Burmese amber provided a detailed structure of their unique proboscis. Surprisingly, a specimen in Spanish amber is carrying clumped pollen that is attributed to a Mesozoic gymnosperm (Peñalver et al., 2015). The co-occurrence of pollen with its insect vector conforms that these long-proboscid insects were gymnosperm pollinators. Zhangsolvids became extinct during the late Cretaceous probably due to the extinction of their gymnosperm food.

## Probable Flies-Angiosperm Associations

Mutualisms between fossil insects and plants are among the most interesting biological associations (Ren et al., 2009; Labandeira and Currano, 2013). Direct evidence of early interactions between insects and their productive organs of plants is that pollen preserved in the guts of fossil insects (Bronstein et al., 2006; Labandeira et al., 2007). Some evidences that specimens with masses of pollen in their guts have been found from the Cretaceous (Krassilov and Rasnitsyn, 1982; Caldas et al., 1989; Huang et al., 2016). Although some pollen grains were found in the guts of several groups, but no record is reported from Mesozoic brachyceran flies till now. Further investigation of brachyceran flies from Cretaceous may provide more evidence.

Very rare definitive evidences of insects carrying pollen grains have been found, such as thrips and dipteran flies found in Early Cretaceous amber of Spain (Peñalver et al., 2012, 2015). The most important indirect evidence for co-evolution of flies and angiosperms may be the mouthparts (Labandeira, 2010). Long mouthparts flies were quite diverse during the Upper Jurassic and Lower Cretaceous, such as Nemestrinidae, Zhangsolvidae, and newly reported the first record of Hilarimorphidae from Lower Cretaceous Lebanese amber (Myskowiak et al., 2016). Mouthparts of fly in mid-Cretaceous Burmese amber also show a high morphological disparity, from thin long to short expanded ones (**Figure 1**). The diversity of proboscis strongly suggests diverse plant hosts (Larson et al., 2001). Modern flower-visiting brachyceran flies usually have long proboscis, such as bee flies and tangle-veined flies. Based on our mid-Cretaceous amber sources, however, tangle-veined flies and bee flies with long proboscis are quite rare, and nearly all specimens have relatively short and expand labellum. Most of these flies in Burmese amber have the labellum consisting of a broad, fleshy expansion that is probably used to feed on nectars, obviously distinct with extant ones that with quite long mouthparts (Grimaldi, 1999). These flies probably obtain nectar from open flowers of various families of plants, and species with longer mouthparts probably feed on deep tubular flowers.

## Conclusion

Tabanidae, Nemestrinidae, Bombyliidae, Eremochaetidae, and Zhangsolvidae had already diversified during or before mid-Cretaceous based on the fossil record and supplementary molecular analyses. Tabanidae, Nemestrinidae, and Bombyliidae currently are among the most common pollinators of angiosperms, and their diversifications are consistent with the rise of angiosperms to widespread floristic dominance. These brachyceran groups probably played an important role in the origin of co-evolutionary relationships with basal angiosperms. Zhangsolvidae and Eremochaetidae became extinction perhaps owing to the Late Cretaceous floral turnover and competition from newly evolved groups. In this regard, the rise of angiosperms not only improved the diversity of flower-visiting flies, but also advanced the turnover and evolution of other specialized flies. Moreover, early reproductive organ-visiting flies (including on those gymnosperms) are responsible for the origin of flowers and the diversity of angiosperms. In this review, we have only scratched the surface of the co-evolution of Cretaceous brachyceran flies with angiosperm, our knowledge of Mesozoic flies-angiosperm mutualisms should greatly expand with more and better preserved fossils and improvements in phylogenetic analysis.

## Author Contributions

BW designed the project; QZ performed the comparative and analytical work, and wrote the manuscript.

## Conflict of Interest Statement

The authors declare that the research was conducted in the absence of any commercial or financial relationships that could be construed as a potential conflict of interest.

## References

[B1] Ansorge J. Mostovski M. B. (2000). Redescription of *Prohirmoneura jurassica* Handlirsch, 1906 (Diptera: Nemestrinidae) from the lower Tithonian lithographic limestone of Eiehstätt (Bavaria). *N. Jb. Geol. Paläont. Mh.* 4 235–243.

[B2] Arillo A. Peñalver E. Pérez-de la Fuente R. Delclòs X. Criscione J. Barden P. M. (2015). Long-proboscid brachyceran flies in Cretaceous amber (Diptera: Stratiomyomorpha: Zhangsolvidae). *Syst. Entomol.* 40 242–267. 10.1016/j.cub.2015.05.062

[B3] Bernardi N. (1973). The genera of the family Nemestrinidae (Diptera: Brachycera). *Arq. Zool.* 24 211–318. 10.11606/issn.2176-7793.v24i4p211-318

[B4] Bernhardt P. Thien L. B. (1987). Self-isolation and insect pollination in the primitive angiosperms: new evaluations of older hypotheses. *Plant Syst. Evol.* 156 159–176. 10.1007/BF00936071

[B5] Bronstein J. L. Alarcón R. Geber M. (2006). The evolution of plant–insect mutualisms. *New Phytol.* 172 412–428. 10.1111/j.1469-8137.2006.01864.x 17083673

[B6] Burger J. F. (2009). “Tabanidae (horse flies, deer flies, tabanos),” in *Manual of Central American Diptera* Vol. 1 eds Brown B. V. Borkent A. Cumming J. M. Wood D. M. Woodley N. E. Zumbado M. A. (Ottawa, ON: NRC Research Press) 495–507.

[B7] Caldas E. B. Martins-Neto R. G. Lima Filho F. P. (1989). *Afropollis* sp. (polém) no trato intestinal de vespa (Hymenoptera: Apocrita: Xyelidae) no Cretáceo da Bacia do Araripe. *Simpósio Geologia Nordeste* 13 195–196.

[B8] Colless D. H. McAlpine D. K. (1991). “Diptera (flies),” in *The Insects of Australia* 2nd Edn Vol. II ed. CSIRO (Melbourne, VIC: Melbourne University Press) 717–786.

[B9] Condamine F. L. Clapham M. E. Kergoat G. J. (2016). Global patterns of insect diversification: towards a reconciliation of fossil and molecular evidence? *Sci. Rep.* 6:19208 10.1038/srep19208 PMC4725974 26778170

[B10] Eggleton P. Belshaw R. (1993). Comparison of dipteran, hymenopteran, and coleopteran parasitoids: provisional phylogenetic explanations. *Biol. J. Linn. Soc.* 48 213–226. 10.1111/j.1095-8312.1993.tb00888.x

[B11] Evenhuis N. L. (1994). *Catalogue of the Fossil Flies of the World (Insecta: Diptera)*. Leiden: Backhuys Publishers.

[B12] Evenhuis N. L. Greathead D. J. (2003). World catalogue of bee flies (Diptera: Bombyliidae): corrigenda and addenda. *Zootaxa* 300 1–64. 10.11646/zootaxa.300.1.1

[B13] Feener D. H. Brown B. V. (1997). Diptera as parasitoids. *Annu. Rev. Entomol.* 42 73–97. 10.1146/annurev.ento.42.1.73 15012308

[B14] Friis E. M. Pedersen K. R. Crane P. R. (2010). Diversity in obscurity: fossil flowers and the early history of angiosperms. *Philos. Trans. R. Soc. Lond. B Biol. Sci.* 365 369–382. 10.1098/rstb.2009.0227 20047865 PMC2838257

[B15] Gilbert F. S. Jervis M. A. (1998). Functional, evolutionary and ecological aspects of feeding-related mouthpart specializations in parasitoid flies. *Biol. J. Linn. Soc.* 63 495–535. 10.1111/j.1095-8312.1998.tb00327.x

[B16] Greathead D. J. Evenhuis N. L. Lamas C. J. E. (2009). “Bombyliidae (bee flies),” in *Manual of Central American Diptera* Vol. 1 ed. Brown B. V. (Ottawa, ON: Library and Archives Canada Cataloguing) 565–576.

[B17] Grimaldi D. Cumming J. (1999). Brachyceran Diptera in Cretaceous ambers and Mesozoic diversification of the Eremoneura. *Bull. Am. Mus. Nat. Hist.* 239 1–124.

[B18] Grimaldi D. A. (1999). The co-radiations of pollinating insects and angiosperms in the Cretaceous. *Ann. Mo. Bot. Gard.* 86 373–406. 10.2307/2666181

[B19] Grimaldi D. A. (2016). Diverse orthorrhaphan flies (Insecta: Diptera: Brachycera) in amber from the Cretaceous of Myanmar: Brachycera in Cretaceous Amber, Part VII. *Bull. Am. Mus. Nat. Hist.* 408 1–131. 10.1206/0003-0090-408.1.1

[B20] Grimaldi D. A. Arillo A. Cumming J. M. Hauser M. (2011). Brachyceran Diptera (Insecta) in Cretaceous ambers, part IV, Significant new Orthorrhaphous taxa. *Zookeys* 148 293–332. 10.3897/zookeys.148.1809 PMC3264415 22287902

[B21] Grimaldi D. A. Barden P. (2016). The Mesozoic Family Eremochaetidae (Diptera: Brachycera) in Burmese amber and relationships of Archisargoidea: Brachycera in Cretaceous Amber, Part VIII. *Am. Mus. Novitates* 3865 1–29. 10.1206/3865.1

[B22] Huang D. Y. Bechly G. Nel P. Engel M. S. Prokop J. Azar D. (2016). New fossil insect order Permopsocida elucidates major radiation and evolution of suction feeding in hemimetabolous insects (Hexapoda: Acercaria). *Sci. Rep.* 6:23004 10.1038/srep23004 PMC4785345 26961785

[B23] Hull F. M. (1973). *Bee Flies of the World: The Genera of the Family Bombyliidae*. Washington, DC: Smithsonian Institution Press.

[B24] Kerr P. H. (2010). Phylogeny and classification of Rhagionidae, with implications for Tabanomorpha (Diptera: Brachycera). *Zootaxa* 2592 1–133.

[B25] Kovalev V. G. (1989). Bremochaetidae, the Mesozoic family of brachycerous dipterans. *Paleontol. J.* 1989 100–105.

[B26] Krassilov V. A. Rasnitsyn A. P. (1982). A unique finding: pollen in the intestine of early Cretaceous sawflies. *Paleontol. J.* 16 80–95.

[B27] Labandeira C. C. (1998). How old is the flower and the fly? *Science* 280 57–59. 10.1126/science.280.5360.57

[B28] Labandeira C. C. (2010). The pollination of mid Mesozoic seed plants and the early history of long-proboscid insects. *Ann. Mo. Bot. Gard.* 97 469–513. 10.3417/2010037

[B29] Labandeira C. C. Currano E. D. (2013). The fossil record of plant-insect dynamics. *Annu. Rev. Earth Planet. Sci.* 41 287–311. 10.1146/annurev-earth-050212-124139

[B30] Labandeira C. C. Kvaèek J. Mostovski M. B. (2007). Pollination fluids, pollen, and insect pollination of Mesozoic gymnosperms. *Taxon* 56 663–695. 10.1073/pnas.1120499109

[B31] Lamas C. J. E. Nihei S. S. (2007). Biogeographic analysis of Crocidiinae (Diptera, Bombyliidae): finding congruence among morphological, molecular, fossil and paleogeographical data. *Rev. Bras. Entomol.* 51 267–274. 10.1590/S0085-56262007000300003

[B32] Larson B. M. H. Kevan P. G. Inouye D. W. (2001). Flies and flowers: taxonomic diversity of anthophiles and pollinators. *Can. Entomol.* 133 439–465. 10.4039/Ent133439-4

[B33] Martins-Neto R. Santos J. (1994). Um novo gênero e uma nova espécie de Mutuca (Insecta, Diptera, Tabanidae) da Formação Santana (Cretáceo Inferior), Bacia do Araripe, Nordeste do Brasil. *Acta Geol. Leopoldensia* 39 289–297.

[B34] Martins-Neto R. G. (2003). The fossil tabanids (Diptera Tabanidae): when they began to appreciate warm blood and when they began transmit diseases? *Mem. Inst. Oswaldo Cruz* 98 29–34. 10.1590/S0074-02762003000900006 12687759

[B35] Mazzarolo L. A. Amorim D. S. (2000). Cratomyia macrorrhyncha, a lower Cretaceous brachyceran fossil from the Santana Formation, Brazil, representing a new species, genus and family of the Stratiomyomorpha (Diptera). *Insect Syst. Evol.* 31 91–102. 10.1163/187631200X00336

[B36] Morita S. I. Bayless K. M. Yeates D. K. Wiegmann B. M. (2016). Molecular phylogeny of the horse flies: a framework for renewing tabanid taxonomy. *Syst. Entomol.* 41 56–72. 10.1111/syen.12145

[B37] Mostovski M. B. (1996). To the knowledge of Archisargoidea (Diptera, Brachycera). Families Eremochaetidae and Archisargidae. *Russ. Entomol. J.* 5 117–124.

[B38] Mostovski M. B. (1998). A revision of the nemestrinid flies (Diptera, Nemestrinidae) described by Rohdendorf, and a description of new taxa of the Nemestrinidae from the Upper Jurassic of Kazakhstan. *Paleontol. J.* 32 369–375.

[B39] Mostovski M. B. Jarzembowski E. A. Coram R. A. (2003). Horseflies and athericids (Diptera: Tabanidae, Athericidae) from the lower Cretaceous of England and Transbaikalia. *Paleontol. J.* 37 162–169.

[B40] Mostovski M. B. Martínez-Delclòs X. (2000). New Nemestrinoidea (Diptera: Brachycera) from the Upper Jurassic-Lower Cretaceous of Eurasia, taxonomy and palaeobiology. *Entomol. Probl.* 31 137–148.

[B41] Myskowiak J. Azar D. Nel A. (2016). The first fossil hilarimorphid fly (Diptera: Brachycera). *Gondwana Res.* 35 192–197. 10.1016/j.gr.2015.05.003

[B42] Nagatomi A. Yang D. (1998). A review of extinct Mesozoic genera and families of Brachycera (Insecta, Diptera, Orthorrhapha). *Entomol. Monthly Mag.* 134 95–192.

[B43] Nel A. (2006). Oldest records of Bombyliidae: Phthiriinae and Mythicomyiidae: Glabellulinae from the Lowermost Eocene amber of France (Diptera: Bombylioidea). *Eur. J. Entomol.* 103 109–114. 10.14411/eje.2006.016

[B44] Nel A. De Ploëg G. (2004). New fossil bee flies (Diptera: Bombylioidea) in the Lowermost Eocene amber of the Paris Basin. *Geol. Acta* 2 57–65.

[B45] Pape T. Blagoderov V. Mostovski M. B. (2011). “Order Diptera Linnaeus, 1758” in *Animal Biodiversity: An Outline of Higher-level Classification and Survey of Taxonomic Richness* ed. Zhang Z.-Q. (Auckland: Magnolia Press) 222–229.

[B46] Peñalver E. Arillo A. Pérez-de la Fuente R. Riccio M. L. Delclòs X. Barrón E. (2015). Long-proboscid flies as pollinators of Cretaceous gymnosperms. *Curr. Biol.* 25 1917–1923. 10.1016/j.cub.2015.05.062 26166781

[B47] Peñalver E. Labandeira C. C. Barrón E. Delclòs X. Nel P. Nel A. (2012). Thrips pollination of Mesozoic gymnosperms. *Proc. Natl. Acad. Sci. U.S.A.* 109 8623–8628. 10.1073/pnas.1120499109 22615414 PMC3365147

[B48] Peralta-Medina E. Falcon-Lang H. J. (2012). Cretaceous forest composition and productivity inferred from a global fossil wood database. *Geology* 40 219–222. 10.1130/G32733.1

[B49] Ren D. (1998). Flower-associated brachycera flies as fossil evidence for Jurassic angiosperm origins. *Science* 280 85–88. 10.1126/science.280.5360.85 9525862

[B50] Ren D. Guo Z. (1995). A new genus and two new species of short-horned flies of Upper Jurassic from northeast China (Diptera: Eremochaetidae). *Entomol. Sin.* 2 300–307. 10.1111/j.1744-7917.1995.tb00051.x

[B51] Ren D. Labandeira C. C. Santiago-Blay J. A. Rasnitsyn A. P. Shih C. K. Bashkuev A. (2009). A probable pollination mode before angiosperms: Eurasian, long-proboscid scorpionflies. *Science* 326 840–847. 10.1126/science.1178338 19892981 PMC2944650

[B52] Rohdendorf B. B. (1968). “New Mesozoic nemestrinids (Diptera, Nemestrinidae),” in *(Jurassic Insects of Karatau)* ed. Rohdendorf B. B. (Moscow: Nauka Press) 180–189.

[B53] Shi G. H. Grimaldi D. A. Harlow G. E. Wang J. Yang M. Lei W. (2012). Age constraint on Burmese amber based on U-Pb dating of zircons. *Cretaceous Res.* 37 155–163. 10.1016/j.cretres.2012.03.014

[B54] Ssymank A. Kearns C. A. Pape T. Thompson F. C. (2008). Pollinating flies (Diptera): a major contribution to plant diversity and agricultural production. *Biodiversity* 9 86–89. 10.1080/14888386.2008.9712892

[B55] Thien L. B. Azuma H. Kawano S. (2000). New perspectives on the pollination biology of basal angiosperms. *Int. J. Plant Sci.* 161 S225–S235. 10.1086/317575

[B56] Ussatchov D. A. (1968). New Jurassic Asilomorpha (Diptera) of the Karatau. *Entomol. Rev.* 47 378–384.

[B57] Wang B. Zhang H. Jarzembowski E. (2013). Early Cretaceous angiosperms and beetle evolution. *Front. Plant Sci.* 4:360 10.3389/fpls.2013.00360 PMC3770909 24062759

[B58] Wedmann S. (2007). A nemestrinid fly (Insecta: Diptera: Nemestrinidae: cf. Hirmoneura) from the Eocene Messel pit (Germany). *J. Paleontol.* 81 1114–1117. 10.1666/pleo06-007.1

[B59] Wedmann S. Yeates D. K. (2008). Eocene records of bee flies (Insecta, Diptera, Bombyliidae, Comptosia): their paleobiogeographic implications and remarks on the evolutionary history of bombyliids. *Palaeontology* 51 231–240. 10.1111/j.1475-4983.2007.00745.x

[B60] Wilkommen J. Grimaldi D. A. (2007). “Diptera: true flies, gnats, and crane flies,” in *The Crato Fossil Beds of Brazil: Window into an Ancient World* eds Martill D. M. Bechly G. Loveridge R. F. (Cambridge: Cambridge University Press) 369–387.

[B61] Winterton S. L. Hardy N. B. Gaimari S. D. Hauser M. Hill H. N. Holston K. C. (2015). The phylogeny of stiletto flies (Diptera: Therevidae). *Syst. Entomol.* 41 144–161. 10.1111/syen.12147

[B62] Woodley N. E. (1989). “Phylogeny and classification of the “orthorrhaphous” Brachycera,” in *Manual of Nearctic Diptera* Vol. 3 ed. McAlpine J. F. (Ottawa, ON: Research Branch, Agriculture Canada) 1371–1395.

[B63] Woodley N. E. (2009). “Nemestrinidae (tangle-veined flies),” in *Manual of Central American Diptera* Vol. 1 ed. Brown B. V. (Ottawa, ON: Library and Archives Canada Cataloguing) 557–560.

[B64] Yeates D. K. (1994). The cladistics and classification of the Bombyliidae (Diptera: Asiloidea). *Bull. Am. Mus. Nat. Hist.* 219 1–191.

[B65] Yeates D. K. (2002). Relationships of extant lower Brachycera (Diptera): a quantitative synthesis of morphological characters. *Zool. Scr.* 31 105–121. 10.1046/j.0300-3256.2001.00077.x

[B66] Zhang J. F. (2012). New horseflies and water snipe-flies (Diptera: Tabanidae and Athericidae) from the Lower Cretaceous of China. *Cretaceous Res.* 36 1–5. 10.1016/j.cretres.2012.01.004

[B67] Zhang J. F. (2014). New male eremochaetid flies (Diptera, Brachycera, Eremochaetidae) from the Lower Cretaceous of China. *Cretaceous Res.* 49 205–213. 10.1016/j.cretres.2014.02.012

[B68] Zhang J. F. Zhang S. Li L. Y. (1993). Mesozoic gadflies (Insecta: Diptera). *Acta Palaeontol. Sin.* 26 595–603.

[B69] Zhang K. Y. Yang D. Ren D. (2014). New short-horned flies (Diptera: Eremochaetidae) from the Early Cretaceous of China. *Zootaxa* 3760 479–486. 10.11646/zootaxa.3760.3.15 24870098

[B70] Zhang K. Y. Yang D. Ren D. Ge F. (2008). New Middle Jurassic tangle-veined flies from Inner Mongolia, China. *Acta Palaeontol. Pol.* 53 161–164. 10.4202/app.2008.0112

[B71] Zhang Q. Q. Zhang J. F. Feng Y. T. Zhang H. C. Wang B. (2016a). An endoparasitoid Cretaceous fly and the evolution of parasitoidism. *Sci. Nat.* 103:2 10.1007/s00114-015-1327-y 26715353

[B72] Zhang Q. Q. Zhang J. F. Wang B. (2016b). A remarkable brachyceran fly (Diptera: Tabanomorpha) from Late Cretaceous Burmese amber. *Cretaceous Res.* 67 1–7. 10.1016/j.cretaes.2016.06.012

[B73] Zhang Q. Q. Zhang J. F. Wang B. (2017). First record of the subfamily Archinemestriinae in the family Nemestrinidae (Diptera: Brachycera) from Upper Cretaceous Burmese amber. *Cretaceous Res.* 75 141–145. 10.1016/j.cretres.2017.03.005

